# Real-Time Prediction of Rate of Penetration in S-Shape Well Profile Using Artificial Intelligence Models

**DOI:** 10.3390/s20123506

**Published:** 2020-06-21

**Authors:** Salaheldin Elkatatny

**Affiliations:** Petroleum Engineering Department, College of Petroleum Engineering & Geosciences, King Fahd University of Petroleum & Minerals, Box: 5049, Dhahran 31261, Saudi Arabia; elkatatny@kfupm.edu.sa

**Keywords:** ROP prediction, artificial intelligence, drilling parameter, S-shape

## Abstract

Rate of penetration (ROP) is defined as the amount of removed rock per unit area per unit time. It is affected by several factors which are inseparable. Current established models for determining the ROP include the basic mathematical and physics equations, as well as the use of empirical correlations. Given the complexity of the drilling process, the use of artificial intelligence (AI) has been a game changer because most of the unknown parameters can now be accounted for entirely at the modeling process. The objective of this paper is to evaluate the ability of the optimized adaptive neuro-fuzzy inference system (ANFIS), functional neural networks (FN), random forests (RF), and support vector machine (SVM) models to predict the ROP in real time from the drilling parameters in the S-shape well profile, for the first time, based on the drilling parameters of weight on bit (WOB), drillstring rotation (DSR), torque (T), pumping rate (GPM), and standpipe pressure (SPP). Data from two wells were used for training and testing (Well A and Well B with 4012 and 1717 data points, respectively), and one well for validation (Well C) with 2500 data points. Well A and Well B data were combined in the training-testing phase and were randomly divided into a 70:30 ratio for training/testing. The results showed that the ANFIS, FN, and RF models could effectively predict the ROP from the drilling parameters in the S-shape well profile, while the accuracy of the SVM model was very low. The ANFIS, FN, and RF models predicted the ROP for the training data with average absolute percentage errors (AAPEs) of 9.50%, 13.44%, and 3.25%, respectively. For the testing data, the ANFIS, FN, and RF models predicted the ROP with AAPEs of 9.57%, 11.20%, and 8.37%, respectively. The ANFIS, FN, and RF models overperformed the available empirical correlations for ROP prediction. The ANFIS model estimated the ROP for the validation data with an AAPE of 9.06%, whereas the FN model predicted the ROP with an AAPE of 10.48%, and the RF model predicted the ROP with an AAPE of 10.43%. The SVM model predicted the ROP for the validation data with a very high AAPE of 30.05% and all empirical correlations predicted the ROP with AAPEs greater than 25%.

## 1. Introduction

The rate of penetration refers to the speed under which the rock is broken under the bit. It is a measure of the progress or speed of drilling per unit area per unit time and reported in feet per hour (ft/hr) in the oil field unit [1]. In essence, a faster rate of penetration is desirable because it is cost-effective, thereby saving a lot of time and resources that could have been expended. However, other problems are associated with a faster rate of penetration. These include poor hole cleaning and excessive vibrations leading to more complications such as losing the bottom hole assembly (BHA) or wellbore instability [2].

The rate of penetration is affected by several factors which are inseparable. These factors have been categorized into five categories and they include formation characteristics, the efficiency of the rig, properties of mud, hydraulic factors, as well as mechanical factors. As asserted by Hossain and Al-Majed [3], these factors can be grouped into controllable and uncontrollable factors. On the one hand, the controllable factors are those that can be altered quickly without necessarily causing significant operational economies such as the design of the bit, the revolution of string per minute, and the weight of bit. On the other hand, the uncontrollable factors are impossible to alter due to geological and economic reasons which include weight and type of mud, underbalanced in formation in pressure, and the size of the bit. As it relates to the properties of fluids and the effects of the rate of penetration, it is hard to alter a single property without causing an impact on others. Therefore, it makes it difficult to assess the real impact of a specific parameter as it relates to the penetration rate [4]. While drilling a well, three components must be considered [5], namely the weight on bit (WOB), drillstring rotation (DSR), and the pumping rate (GPM) [6].

To help determine the effects of drilling parameters, as well as the properties of mud, on the penetration rate, there are established methods that have been proven to be effective in doing so. Among these established methods are included the basic mathematical and physics equations which help derive the relationships between these items [7]. In addition, the use of correlation and connecting them to obtain such relations has proven to be critical. However, there is no reliable or solid method that currently exists due to the level of complexity associated with the process of drilling. It is also not easy to capture each factor for predicting the penetration rate. Ricardo et al. [8], posited that there was no complete model that could be used to capture all parameters during drilling, as well as the properties of mud which affect the rate of penetration. Therefore, it is important to treat them independently and come up with individual correlations for reliable results.

The S-shape well profile undergoes a build, hold, and then drop section [9]. The well begins as vertical, then at the desired depth, directional BHA is picked to build an angle, then, hold it until a predefined departure is achieved, and then the angle is dropped back to zero [10]. In the upcoming sections, rotary BHA is used, however, when the horizontal displacement is very far, then, a performance motor is selected to allow downhole rotation. This reduces the surface DSR due to torque limitation. In addition, a casing protector needs to be used to reduce casing wear [11]. This profile is selected if there is a surface location such as having a mountain above the reservoir or to get away from subsurface trouble zones such as fractures or salt domes [3]. Different formations are drilled across the tangent which can cause wellbore stability issues across some formations. The sidewall forces keep changing and cycling depending on the BHA position. Torque and drag are present as well, causing some forces to be lost such as vertical forces (WOB) and rotational forces (DSR). Hole cleaning is a big challenge since the cuttings can easily avalanche downward [12,13].

### Effect of Drilling Parameters on the Rate of Penetration

Maurer [14] adopted the concept of optimum hole cleaning and derived his rate of penetration (ROP) model for tricone bit type. He assumed that cuttings were being removed as the bit tooth impacted the formation rock. Bingham [15] performed multiple laboratory experiments in order to develop his ROP model. He assumed that the weight on bit threshold could be neglected. This resulted in having the ROP as a function of the rotary speed (DSR) and applied weight on bit. In his model, he developed a WOB exponent which could be calculated through extermination. Bourgoyne and Young [16] presented a mathematical regression model that used existing drilling data to calculate multiple exponents that were required for developing the full model. Each exponent captured a certain physical or mechanical meaning for the drilling process such as the effect of overbalance, overburden pressure, and bit tooth wear. Warren [17] developed his model considering an optimum cleaning scenario for tricone bit type, where the removal of cuttings rate under the bit equaled the rate of generating new cuttings. Al-AbdulJabbar [18] developed a new ROP model and took into consideration drilling mechanical and hydraulic parameters, as well as mud properties. Using nine inputs, two exponents were calculated which were bit exponent and formation compressive strength. Each formation type had its compressive strength coefficient.

Different previous studies have suggested the use of artificial intelligence (AI) to improve the predictability of different parameters related to the oil industry [19,20,21,22,23,24,25,26,27]. Bilgesu et al. [28] suggested the use of AI techniques for ROP prediction. They developed two artificial neural networks (ANN) models for predicting ROP, while drilling through various nine formations in different vertical wells. Amar and Ibrahim [29] developed two ANN models to evaluate the ROP based on the formation depth, ECD, WOB, DSR, pore pressure gradient, drill bit’s tooth wear, and Reynolds number function. A comparison of the prediction power of the developed ANN-based models with the available empirical equations showed that both ANN-based models were highly accurate for estimating the ROP as compared with the empirical equations.

Elkatatny [30] used the ANN feedforward network to predict ROP on three wells. Using two wells, the model was trained on 3333 data points with a correlation coefficient of 0.99 and an average absolute percentage error of 5%. Then, using 2700 unseen data from the third well, the model was able to predict the rate of penetration with a correlation coefficient of 0.99 and an average absolute percentage error of 4%. Al-AbdulJabbar et al. [31] used a feedforward ANN to predict ROP on three well. Using 1500 data points from only single well, the model was able to predict the rate of penetration with a correlation coefficient of 0.92. Later on, the model was used to predict the other two wells with unseen data with a correlation coefficient of 0.95 and 0.94, respectively. Only one well was used in building the model which showed the power of AI in modeling and prediction. Elkatatny et al. [32] demonstrated that once the ANN model was optimized and an empirical correlation was developed, the model could be converted from a black box to a white box making it flexible to deploy in real field applications and environments. Ahmed et al. [33] developed a rate of penetration support vector machine AI model. Using 10 inputs representing drilling mechanical parameters and fluid properties, he developed a resilient ROP model with an AAPE of 2.83%. Al-AbdulJabbar et al. [34] used ANN coupled with self-adaptive differential evolution (SaDE) to predict ROP in horizontal carbonate reservoirs. Using six inputs that coupled drilling mechanical parameters and formation petrophysical properties, such as gamma ray, resistivity, and bulk density, he achieved a strong correlation coefficient of 0.96 and an AAPE of 5.12% after building the model. Using another well with unseen data, he obtained R and AAPE values of 0.95 and 5.8% respectively. The ROP model was turned from a black box to a white box through extracting the weights and biases in a matrix form. A summary of these models including inputs and equations are presented in Table A1, Appendix B.

The main objective of this paper was to build new ROP models for the first time for the S-shape well profile based on the optimized fuzzy inference system (ANFIS), functional neural networks (FN), random forests (RF), and support vector machine (SVM). These models were built to enable a real-time ROP estimation based on the obtained data from the rig real-time sensors such as WOB, DSR, SPP, GPM, and T.

## 2. Artificial Intelligence Models Theory

ANFIS is the first model used in this study which is a fuzzy subtractive clustering-based fuzzy inference system. The fuzzy inference system consists of a multilayer feedforward adaptive network, and in this network, a specific function is applied to the incoming signal through the training nodes. The model training is conducted in the following two stages: First, the forward pass where the functional signals of the input training data going forward and the parameter in the output is identified through the least square formula, and secondly, the backward pass where the input parameters are updated using the gradient method while the error rates propagate in the opposite [35].

FN is the second model used in this work; this model is very similar to the usual ANN model, but it uses a generalized functional model while ANN uses the sigmoidal common model. In addition, the neuron’s functions of the FN are learned based on the existing training data, and this means the weights associated with these neurons are not needed [36]. The FN model is also characterized by the presence of different arguments in neural functions as compared with the ANN which has one argument [37].

The third model considered, in this study, is the RF which was developed to perform classification and regression tasks [38]. This model combines hundreds of decision trees; every decision tree is trained on different observations, every tree consists of several nodes, and every node considers several features. Then, the final prediction of the random forest model is defined as the average of every tree [39]. 

The last artificial intelligence model considered in this study is the SVM which was developed earlier in the framework of statistical learning theory as a classifying algorithm. This model uses a multidimensional hyperplane that helps to divide or classify the data into two or more divisions based on the kernel, margin, gamma, and the regularization parameter (C) [40].

## 3. Data Overview and Preparation

In this work, the field data were obtained from three S-shape wells that share the same hole size and intersect the same geological lithology. These wells were drilled using directional BHA and measurement while drilling (MWD). All the well profiles started as vertical, then, the section was kicked off until the tangent section was reached. The tangent was held at ±25–30°, and then the well was dropped back to zero. The surface data were acquired through real-time sensors, which were recorded on a footage base.

**Figure 1:**
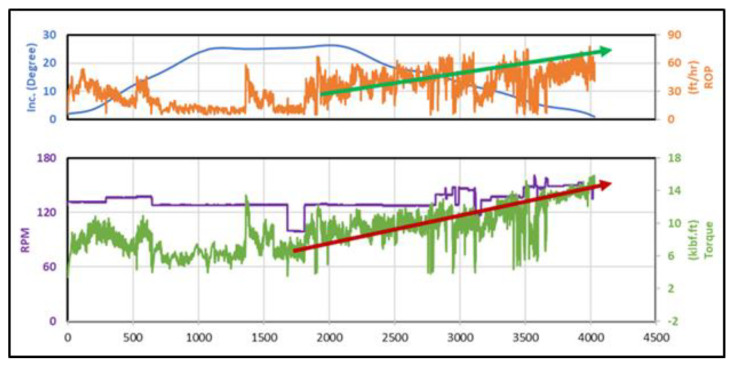
The correlation coefficient between the rate of penetration (ROP) and the training parameters.

It is very important to recognize that the MWD tool provides information about inclination and azimuth based on a mud plus system which requires no circulation during the measurement. The MWD data are transferred to the surface approximately every 100 ft, while the connection time for the drill pipe stands. Therefore, it is not recommended to include the inclination data as an input parameter to be able to predict the ROP on a footage basis. The geological data, which are obtained from the logging while drilling (LWD) tool, was not available in this study, because it is not common to run the LWD tool outside the reservoir section. At the same time, the changes in pipe speed and torque data compensate for the effect of the geological data on the ROP prediction. The inclination data which is available every 100 ft (this is not a real-time record as the drilling mechanical parameters) did not affect the accuracy of the developed models for ROP prediction. This was confirmed, since the accuracies of the developed models without including the inclination were very high, which could be explained, as shown in Figure 1, by the fact that the change in the ROP took place even when the inclination was constant as compared with the torque which changed consistent with the change of the ROP.

The collected data initially included all types of drilling operations performed in the 12 inches section which included tripping, drilling, and deploying casing. The important and necessary drilling data that were used included weight on bit (WOB), drillstring rotation (DSR), torque (T), pumping rate (GPM), and standpipe pressure (SPP) [18].

Data cleaning was conducted to remove all unrealistic values and outliers. The first step was only to extract the portion where new drilling footage was made while discarding the remaining data, which required a human interface using data filtering and elimination. The second step was to clean the data based on the standard deviation, where all the data values without the range of ±3.0 standard deviation were removed from the data. Although the rotary steerable system (RSS) was used, somehow, the data were noisy. The RSS drive resulted in a much smoother hole as compared to using a mud motor. However, different formations were being drilled with ±25–30° inclination, which caused wellbore stability issues across some formations. In addition, sidewall forces kept changing and cycling depending on the BHA position. This introduced vibrations to the BHA, as well as excessive torque and drag. All of these affected the data quality, especially when the well inclination was approaching zero from its maximum value.

**Figure 2:**
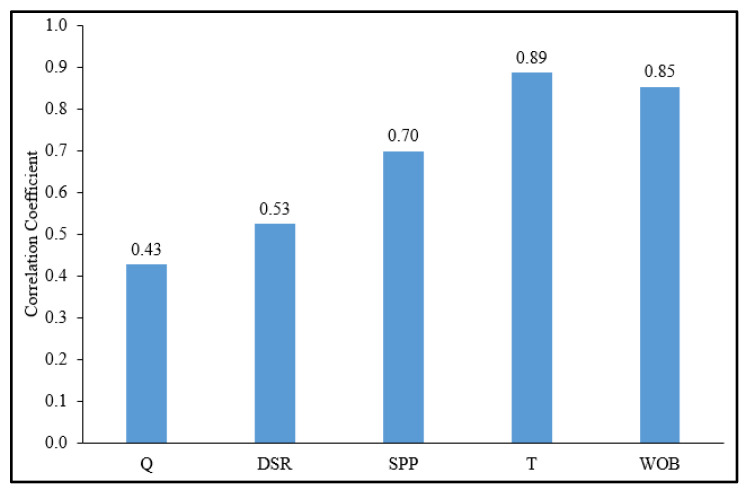
The correlation coefficient between the ROP and the training parameters.

Figure 2 compares the correlation coefficients (R) among the ROP and all training parameters after the cleaning process. As indicated in this figure, the ROP is strongly affected by the T and WOB with Rs of 0.89 and 0.85, respectively, and the ROP has moderate functions with Q, DSR, and SPP with Rs of 0.43, 0.53, and 0.70, respectively.

At the end of a data cleaning process, the data consists of 4012, 1717, and 2500 data points from Well A, Well B, and Well C. Table 1 shows the statistical analysis for the training data obtained from Well A and Well B after the cleaning process. As indicated in this table, Q is between 559 and 993 GPM, DSR is ranging from 99 to 159 rpm, SPP is from 912 to 2490 psi, T is between 4.11 and 15.9 klbf-ft, WOB is from 5.14 to 57.2 klbf, and ROP is ranging from 6.34 to 64.5 ft/hr.

## 4. Artificial Intelligence Models Optimization

**Figure 3:**
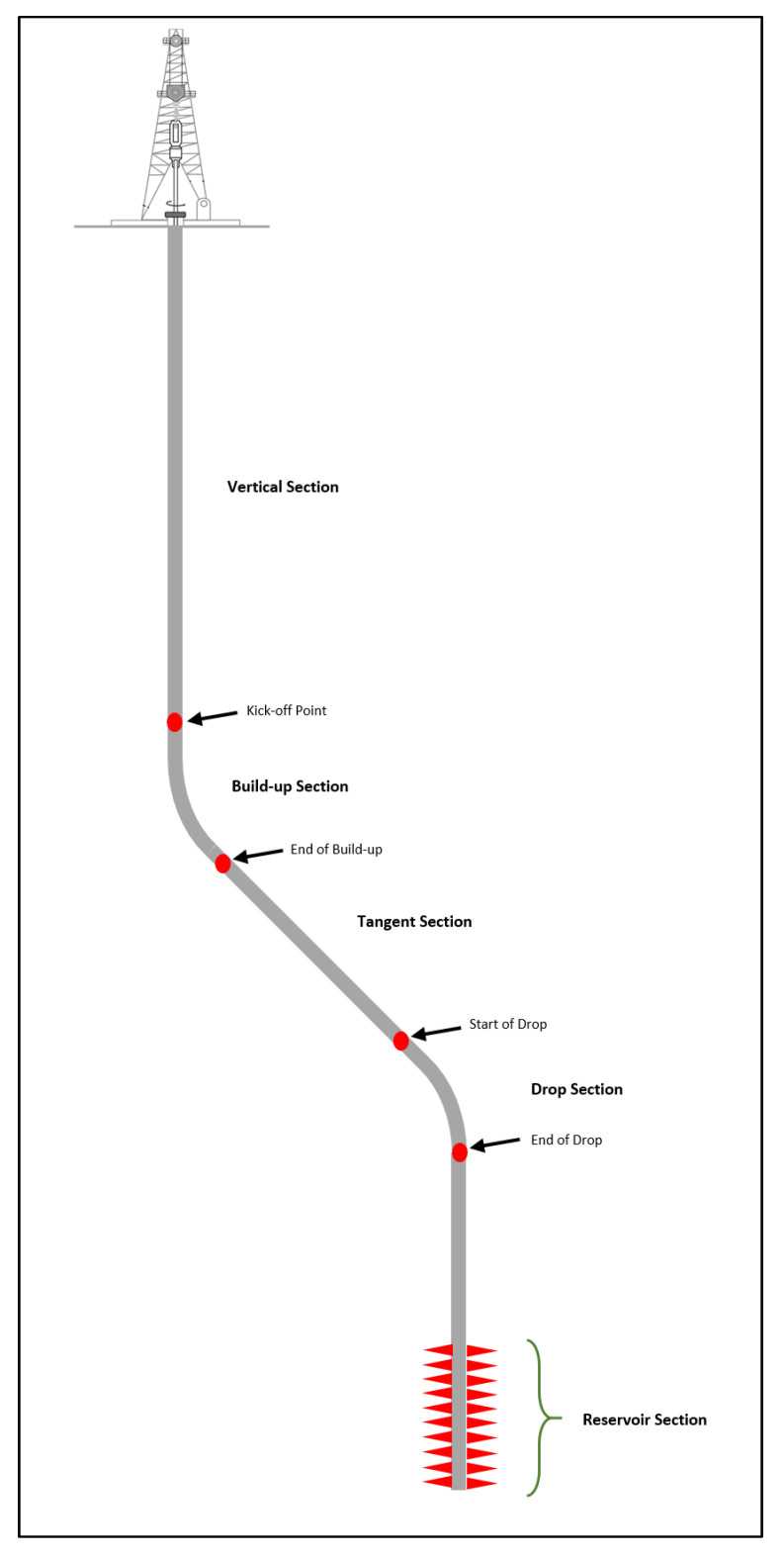
The schematic of the S-shape well trajectory.

Since the S-Shape profile, with the schematic shown in Figure 3, had dual inclination phenomena (build then drop), two wells were used for training (Well A and Well B), and one for validation (Well C) with a ratio of 2.29:1. The two wells with the highest and lowest curvature were used to predict the well in between. The AI models were built using the ANFIS, FN, RF, and SVM models which were trained using the drilling parameters of the weight on bit (WOB), drillstring rotation (DSR), torque (T), pumping rate (GPM), and standpipe pressure (SPP). The data were loaded into the AI modeling software as inputs and output vectors separately. The data were randomly divided into a 70:30 ratio to be used in the training and testing stages. Well A and Well B were combined in the training-testing phase, where multiple AI model design parameters were varied on a trial-and-error basis to achieve the optimum prediction of the rate of penetration with the least amount of error. The design parameters of the ANFIS model optimized during this step were cluster radius and number of iterations, whereas, for the FN model, the design parameters of the training method and training function type were optimized. The maximum depth and maximum features of the RF model and the kernel, gamma, number of iterations, and C parameter of the SVM were optimized, in this study, to improve the ROP prediction. The governing factors for the selection of the optimum parameters for the AI models were the correlation coefficient (R) and the average absolute percentage error (AAPE), which are defined in Appendix A.

Table 2 summarizes the optimized artificial intelligence models. As indicated in this table, the optimized ANFIS model has a cluster with 0.1 radius and 300 iterations. The FN model has a training method and function type of nonlinear function without iteration terms and forward selection method, respectively. The optimized RF model is characterized by a maximum depth of 29 and maximum features of sqrt. The optimum design parameters of the SVM model are the radial basis function kernel, gamma scale, 1000 iterations, and C of 100.

Using the optimized parameters for the ANFIS-ROP model, sensitivity analysis was performed to evaluate the effect of including the inclination as an input parameter. Table 3 shows that for the training and testing data, there was little effect of including the inclination as an input parameter, where there was an increase of the AAPE from 9.57% to 13.81%, whereas for the validation data there was a significant negative effect of including the hole inclination as an input where the error was increased from 9% to 51%. On the basis of this sensitivity, it was decided to exclude the inclination from the input parameters. Please note that interpolation was performed to have a complete profile for the inclination as it was recorded every 100 ft.

## 5. Results and Discussion

### 5.1. Training the Artificial Intelligence Models

Drilling parameters were used as dependent parameters for the rate of penetration prediction using the ANFIS, FN, RF, and SVM models. The developed models were trained using the drilling parameters of the Q, DSR, SPP, T, and WOB.

**Figure 4:**
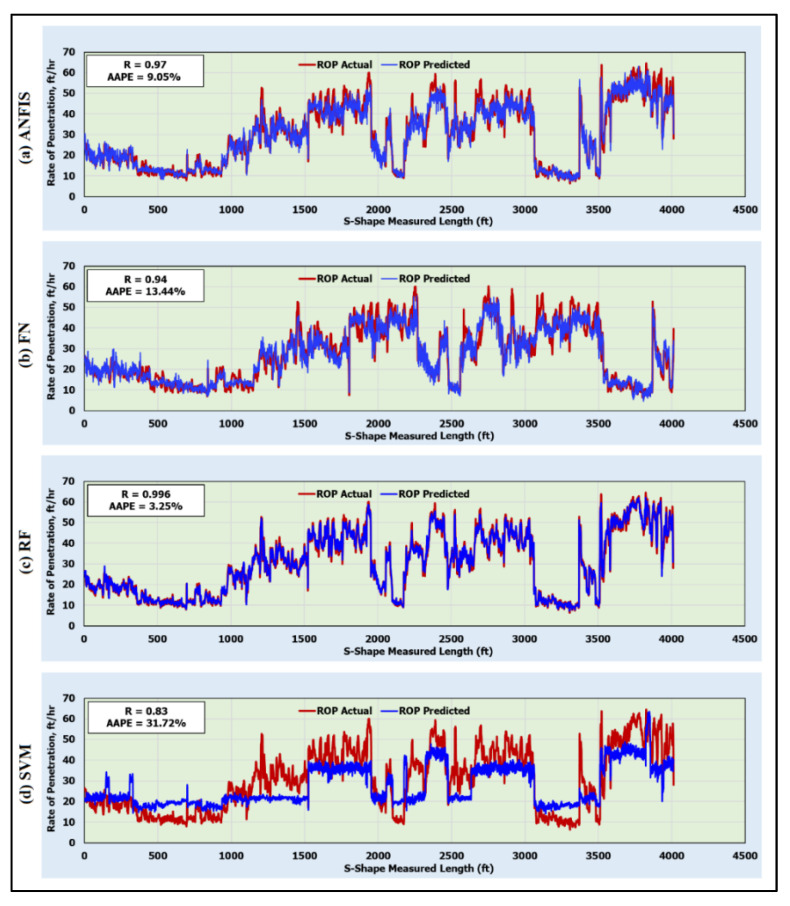
Predicted and actual ROP along the S-shape section for the training data (4012 data points) using (**a**) ANFIS; (**b**) functional neural networks (FN); (**c**) random forests (RF); and (**d**) support vector machine (SVM) models.

Figure 4 shows the prediction of the rate of penetration by the developed AI models for the training data. The results showed correlation coefficients (Rs) of 0.97, 0.94, 0.996, and 0.83 between the actual and predicted ROP with average absolute percentage errors (AAPEs) of 9.50%, 13.44%, 3.25%, and 31.72% for the ANFIS, FN, RF, and SVM models, respectively. These results show that the ANFIS, FN, and RF models’ performances were very satisfactory, whereas the SVM. model was not able to accurately predict the ROP.

### 5.2. Testing Artificial Intelligence Models

**Figure 5:**
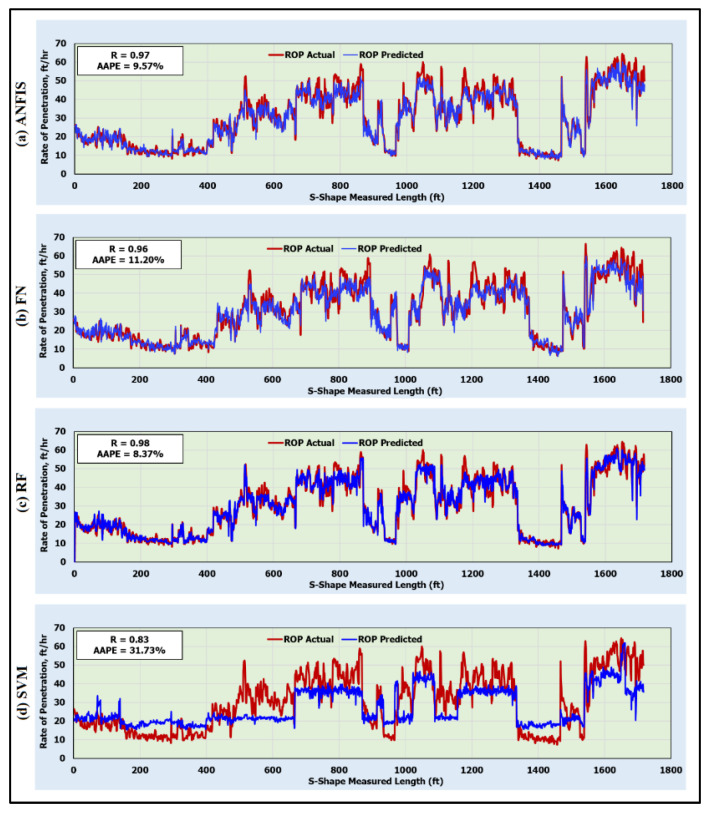
Predicted and actual ROP along the S-shape section for the testing data (1717 data points) using (**a**) ANFIS; (**b**) FN; (**c**) RF; and (**d**) SVM models.

The optimized ANFIS, FN, RF, and SVM models were, then, tested on 1717 data points. Figure 5 shows the prediction of the rate of penetration by the developed AI models for the testing data. These results confirmed the high accuracy of the optimized AI models in estimating the ROP for the testing data. The ANFIS model predicted the ROP with an R of 0.97 and an AAPE of 9.57%. The FN model predicted the ROP with an R and AAPE of 0.96 and 11.20%, respectively. The RF model was the best to estimate the ROP for the testing dataset with an R and AAPE of 0.98 and 8.37% (Figure 5c), respectively, whereas the SVM model predicted the ROP with the least accuracy among the other models. The SVM model estimated the ROP with an R of 0.83 only and a very high AAPE of 31.73% (Figure 5d).

### 5.3. Validating the Artificial Intelligence Models and Comparison with the Published ROP Correlations

**Figure 6:**
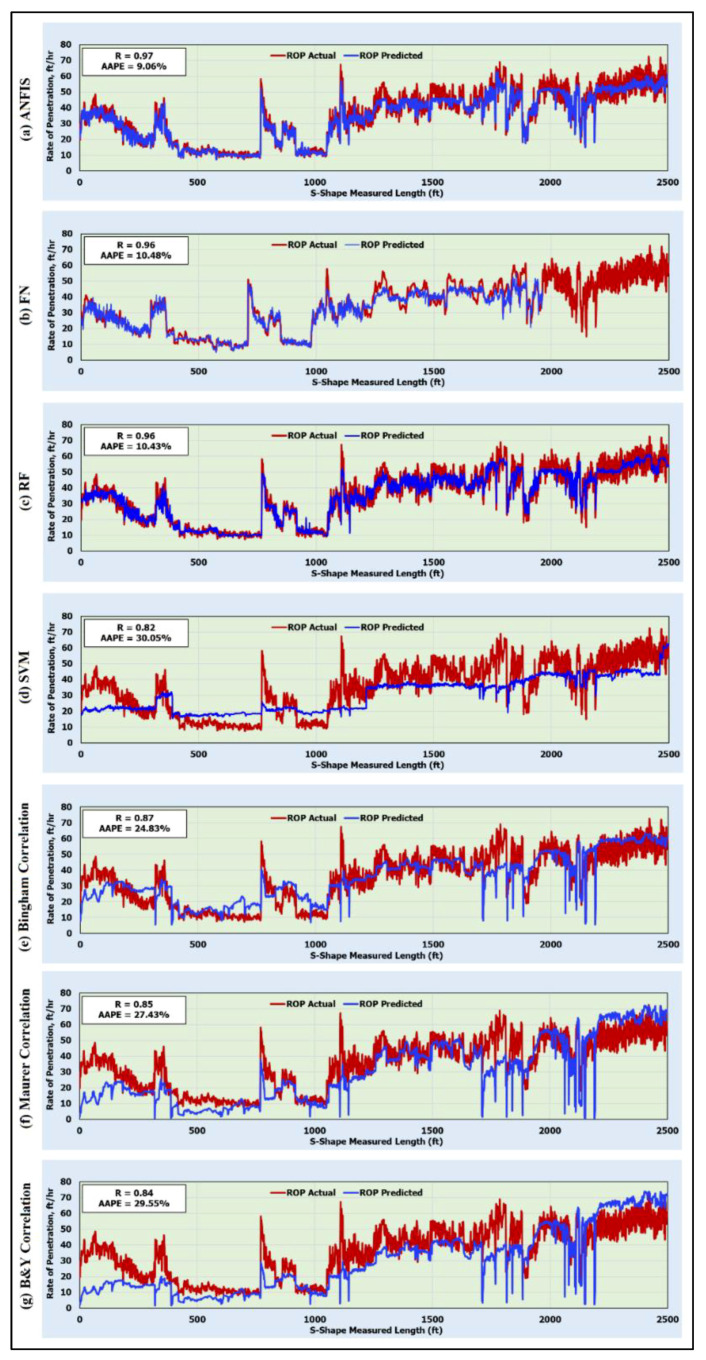
Predicted and actual ROP along the S-shape section for the validation data (2500 unseen data points) using (**a**) ANFIS; (**b**) FN; (**c**) RF; (**d**) SVM; (**e**) Bingham correlation; (**f**) Maurer correlation; and (**g**) Bourgoyne–Young correlation.

To validate the developed AI models, and ensure that they did not memorize the data, Well C was used to predict the rate of penetration with more than 2500 unseen data points. Figure 6 compares the predicted versus an actual rate of penetration for the validation data. The ANFIS model overperformed all AI models developed in this study and all other correlations in estimating the ROP for the validation data collected from Well C. The R and AAPE for estimating the ROP using the ANFIS model were 0.97 and 9.06%, respectively. The FN model also overperformed the previously developed empirical correlations for estimating the ROP with an R of 0.96 and an AAPE of 10.48%. The RF model also overperformed all correlations for estimating the ROP for the validation data where it predicted the ROP with an R of 0.96 and an AAPE of 10.43%. Whereas the SVM model predicted the ROP for the validation data with very low accuracy in terms of an R and AAPE of 0.82 and 30.05% (Figure 6), respectively.

**Figure 7:**
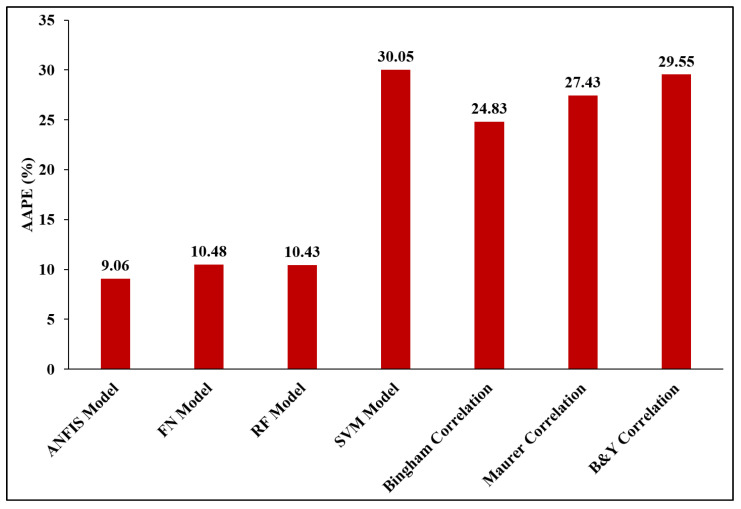
Comparison of the AAPE for ROP estimated using the ANFIS, FN, RF, and SVM models, as well as Bingham correlation, Maurer correlation, and Bourgoyne–Young correlation for the validation data.

All available empirical correlations predicted the ROP with very high AAPEs and low Rs. Bingham, Maurer, and Bourgoyne–Young correlations estimated the ROP with Rs of 0.87, 0.85, and 0.84 and AAPEs of 24.83%, 27.43%, and 29.55%, respectively. These results confirmed the high accuracy of the developed AI models as compared with the available empirical correlations. The results in Figure 7 show the comparison of the AAPEs for the validation data, and confirms the high accuracy of the developed ANFIS, FN, and RF models as compared with the SVM model and other available empirical correlations. Table A2 (Appendix C) shows the comparison of the numerical values of the ROP estimated using the empirical correlations with the ROP values estimated with the AI models for 20 data points selected randomly from Well C. The values reported in this table confirm that the values predicted with the ANFIS, FN, and RF models are closer to the actual ROP as compared with the values predicted using the SVM model or the empirical correlations.

## 6. Conclusions

Actual field data from three S-shape wells were used for the rate of penetration prediction using AI models of the ANFIS, FN, RF, and SVM based on five inputs of the DSR, SPP, Q, T, and WOB. On the basis of the results, the following can be concluded:

- The ANFIS, FN, and RF models could effectively predict the ROP from the drilling parameters in the S-shape well profile for training, testing, and validation data, whereas the SVM model showed very low accuracy in estimating the ROP.
- The developed ANFIS, FN, and RF models predicted the ROP with AAPEs of 9.50%, 13.44%, and 3.25%, respectively, for the training data.
- For the testing data, the optimized ANFIS, FN, RF models estimated the ROP with AAPEs of 9.57%, 11.20%, and 8.37%, respectively.
- The developed ANFIS, FN, and RF models outperformed the SVM model and the three published empirical correlations for estimating the ROP for the validation data.

## Figures and Tables

**Table 1:** Well A and Well B training data statistical parameters (model training data).

Parameter	Q (gpm)	DSR (rpm)	SPP (psi)	T (klbf-ft)	WOB (klbf)	ROP (ft/h)
Minimum	559	99	912	4.11	5.14	6.34
Maximum	993	159	2490	15.9	57.2	64.5
Range	434	60	1579	11.8	52.1	58.1
Standard deviation	94.0	10.8	347	2.26	11.4	15.0
Skewness	−0.596	−0.976	−0.349	0.524	0.045	0.184
Kurtosis	−0.692	2.643	−0.952	−0.140	−0.941	−1.193

**Table 2:** The optimized artificial intelligence models.

ANFIS	
Cluster radius	0.1
Ep-size	300
**FN**	
Training method	Nonlinear function without iteration terms
Training function type	Forward selection method
**RF**	
Maximum depth	29
Maximum feature	Sqrt
**SVM**	
Kernel	Radial basis function
Gamma	Scale
No. of iterations	1000
C	100

**Table 3:** Effect of including the hole inclination as an input parameter using the optimized fuzzy inference system (ANFIS).

	Training		Testing		Validation	
	R	AAPE %	R	AAPE %	R	AAPE %
Without inclination	0.97	9.05	0.97	9.57	0.97	9.06
With inclination	0.94	12.85	0.92	13.81	0.16	51.29

## Abbreviations

ANN	Artificial neural networks
AAPE	Average absolute percentage error
AI	Artificial intelligence
BHA	Bottom hole assembly
FN	Functional neural networks
GPM	Gallon per minute
GR	Gamma ray
LWD	Logging while drilling
MD	Measured depth
MW	Mud weight
MWD	Measurement while drilling
MFV	Marsh funnel viscosity
PV	Plastic viscosity
Q	Flow rate
R	Correlation coefficient
RF	Random forests
RMSE	Root mean square error
ROP	Rate of penetration
DSR	Drillstring rotation
SaDE	Self-adaptive differential evolution
SPP	Standpipe pressure
SVM	Support vector machine
TFA	Total flow area
TVD	True vertical depth
UCS	Unconfined compressive strength
WOB	Weight on bit

## Appendix A

The correlation coefficient (*R*) and the average absolute percentage error (*AAPE*) are defined by the following equations, respectively:

(A1) $$R=\sqrt{1-\frac{\sum_{i}^{N}{\left(y_{iact}-y_{ipred}\right)}^{2}}{\sum_{i}^{N}{\left(y_{iact}\right)}^{2}-\frac{\sum_{i}^{N}{\left(y_{ipred}\right)}^{2}}{N}}}$$

(A2) $$AAPE=\frac{1}{N}\overset{N}{\underset{i=1}{\sum }}\frac{\left|y_{iact}-y_{ipred}\right|}{y_{iact}}$$

where *y_iact_* is the actual value, *y_ipred_* is the predicted value, and *N* is the number of data points. Higher accuracy and better performance of prediction are indicated by higher values of *R* and lower values of the root mean square error (RMSE).

## Appendix B

The table below summarizes multiple published ROP models including ones that were developed empirically or using artificial intelligence techniques.

**Table A1:** Summary of previous ROP models.

Reference	Method	Input Parameters	Formula	Constants
Maurer [14]	Empirical	*DSR*, *WOB*, bit size, uniaxial compressive strength (*UCS*)	$R=k\frac{NW^{2}}{D^{2}S^{2}}$	*K* = 60,087 × 10^3^
Bingham [15]	Empirical	*DSR*, *WOB*, bit size	$R=K{\left(\frac{W}{d_{b}}\right)}^{a}N^{c}$	*K* = 1.04; *a* = 0.081; *c* = 1.08
Bourgoyne and Young’s [16]	Empirical	True vertical depth (*TVD*), mud weight (*MW*), pore pressure, *WOB*, *DSR*, bit wear, Q, *TFA*	$\frac{d}{dt}\left(R\right)=e^{\left(a_{1}+\sum_{i=2}^{8}a_{i}x_{i}\right)}$	*a_1_* = 14.35; *a_2_* = −1.5 × 10^−3^; *a_3_* = −9 × 10^−4^; *a_4_* = 3 × 10^−4^; *a_5_* = 1.496; *a_6_* = 1.269; *a_7_* = 0.118
Warren [17]	Empirical	*WOB*, *DSR*, bit size, *UCS*	$R={\left[\frac{aS^{2}d_{b}^{2}}{N^{b}W^{2}}+\frac{c}{Nd_{b}}\right]}^{-1}$	
Osgouei [4]	Empirical	TVD, *MW*, pore pressure, *WOB*, DSR, bit wear, *Q, TFA*, bit type, inclination	$\frac{d}{dt}\left(R\right)=e^{\left(a_{1}+\sum_{i=2}^{11}a_{i}x_{i}\right)}$	
Al-AbdulJabbar [18]	Empirical	*DSR*, *WOB*, torque, *SPP*, *Q*, bit size, *UCS*, *MW*, drilling fluid plastic viscosity (*PV*)	$ROP=\frac{WOB^{a}\times RPM\times T\times SPP\times GPM}{d_{b}^{2}\times \rho \times PV\times UCS^{b}}$	
Elkatatny et al. [41]	ANN	*Q*, *SPP*, *DSR*, *WOB*, *MW*, torque, *PV*	-	
Al-AbdulJabbar et al. [31]	ANN	*WOB*, *DSR*, *SPP*, *Q*, torque	-	
Ahmed et al. [33]	SVM	*Q*, *SPP*, *DSR*, *WOB*, *MW*, torque, drilling fluid yield point, marsh funnel viscosity, *PV*, solids volume	-	
Al-AbdulJabbar et al. [34]	ANN SaDE	*DSR*, torque, *WOB*, *GR*, formation deep resistivity, formation bulk density	-	

## Appendix C

**Table A2:** Shows the main input parameters for ROP prediction using previous empirical correlations, and the actual and predicted ROP values.

S-Shape Measured Length (ft)	Inputs									Actual ROP, ft/hr	* Empirical Correlations-Based ROP, ft/hr			AI-Based ROP, ft/hr			
	GPM	RPM	WOB, Klbf	Torque, Klbf-ft	SPP, psi	Density, PCF	Bit Size, in	PV	UCS, psi		Bingham [15]	Maurer [14]	B&Y [16]	ANFIS	FN	RF	SVM
116	843.91	131	32.46	10.4	1715.77	67	12	30	35,000	36.04	32.06	23.04	16.77	36.75	36.84	36.63	23.42
133	848.33	132	31	9.88	1704.99	67	12	30	35,000	34.24	30.81	21.17	15.87	33.91	32.81	36.62	22.52
165	838.79	131	32.81	8.03	1667.65	67	12	30	35,000	26.02	32.42	23.54	17.21	26.43	23.76	28.26	22.02
241	843.76	136	26.39	7.32	1708.81	67	12	30	25,000	19.25	26.93	15.81	13.36	19.95	18.55	20.22	21.74
244	841.18	136	26.26	8.29	1706.76	67	12	30	25,000	24.22	26.79	15.65	13.27	24.25	22.99	25.14	21.88
249	844.89	137	25.79	7.82	1697.29	67	12	30	25,000	17.88	26.50	15.21	13.05	17.88	20.40	20.89	21.28
254	845.49	136	26.66	7.74	1681.81	67	12	30	25,000	20.69	27.21	16.13	13.60	20.82	20.02	20.65	20.93
288	844.31	136	27.62	7.31	1675.32	67	12	30	25,000	19.61	28.23	17.32	14.48	18.64	18.45	20.11	20.96
306	843.33	136	27.67	8.26	1690.02	67	12	30	25,000	22.85	28.28	17.38	14.57	23.89	23.03	23.11	21.56
362	940.81	137	27.31	8.2	2027.27	67	12	30	30,000	23.07	28.12	17.05	14.86	24.13	24.57	23.63	28.72
365	948.98	138	27.66	8.34	2050.49	67	12	30	30,000	23.5	28.72	17.62	15.31	23.12	25.55	22.02	29.39
388	917.14	136	28.79	6.57	1961.94	67	12	30	30,000	18.09	29.47	18.81	15.97	17.52	17.83	18.86	27.45
1076	698.38	128	31.46	8.3	1371.99	67	12	30	25,000	27.61	30.27	21.14	19.81	26.69	25.08	26.23	21.28
1326	892.46	128	41.82	8.5	2077.77	67	12	30	25,000	35.83	40.66	37.36	33.28	37.25	35.17	36.01	36.55
1488	898.03	128	38.49	7.95	2065.37	67	12	30	25,000	33.81	37.31	31.65	30.48	33.70	30.70	34.64	34.88
1720	887.69	128	30.47	11.38	2040.27	67	12	30	25,000	42.82	29.28	19.84	22.41	40.92	46.10	43.66	32.95
1723	890.21	140	34.16	10.45	2060.76	67	12	30	25,000	44.91	36.31	27.27	29.83	44.08	43.12	48.13	34.97
1729	891.19	140	35.53	10.89	2048.1	67	12	30	25,000	49.96	37.82	29.50	31.67	48.33	46.20	54.55	34.72
1827	856.28	128	36.48	9.27	2058.21	67	12	30	25,000	37.92	35.29	28.43	29.95	36.92	37.98	37.87	36.65
1831	851.57	146	37.61	9.47	2075.92	67	12	30	25,000	48.23	41.97	34.47	37.20	46.08	41.28	47.47	38.81

***** The inputs for Bingham, RPM and WOB; for Maurer, RPM, WOB, UCS, and bit size; for Bourgoyne–Young, RPM, WOB, UCS, bit size, GPM, torque, SPP, density, and PV.
